# Dexmedetomidine Versus Fentanyl on Time to Extubation in Patients with Morbid Obesity Undergoing Laparoscopic Sleeve Gastrectomy

**DOI:** 10.5812/aapm-144776

**Published:** 2024-05-15

**Authors:** Doha Mohammed Bakr, Rasha Behery Youssef, Maged Salah Mohamed, Moataz Salah Khalil

**Affiliations:** 1 Anesthesiology, Department of Surgical Intensive Care and Pain Management, Faculty of Medicine, Helwan University, Helwan, Egypt; 2 Anesthesiology, Surgical Intensive Care and Pain Management, Faculty of Medicine, Cairo University, Cairo, Egypt

**Keywords:** Dexmedetomidine, Fentanyl, Extubation, Morbid Obesity, Laparoscopic Sleeve Gastrectomy

## Abstract

**Background:**

Sleeve gastrectomy (SG) is an effective method for managing obesity. While opioids are used for their hemodynamic stability and their ability to reduce intraoperative stress, they also have reported side effects. Dexmedetomidine (DEX), an α2 adrenergic receptor agonist, is noted for its analgesic and anesthetic-sparing effects, leading to a higher quality of recovery.

**Objectives:**

The study aims to compare the effects of fentanyl and dexmedetomidine (DEX) on the recovery of morbidly obese patients following laparoscopic sleeve gastrectomy (SG).

**Methods:**

This randomized, double-blind study involved 64 patients, equally divided into two groups. The Dexmedetomidine group (Group D) received an intravenous (IV) loading dose of dexmedetomidine (1 μg/kg) over 15 minutes before anesthesia induction, followed by a 10 mL saline 0.9% infusion over 60 seconds during induction. Post-intubation, dexmedetomidine was administered at 0.5 μg/kg/h. The Fentanyl group (Group F) received a volume-matched saline 0.9% IV over 15 minutes pre-induction and fentanyl (1 μg/kg) diluted in 10 ml saline 0.9% IV over 60 seconds during induction. After intubation, a continuous fentanyl infusion was maintained at a rate of 1 μg/kg/hr.

**Results:**

Extubation time was significantly shorter in the Dexmedetomidine group (Group D) at 8.25 ± 2.7 minutes compared to the Fentanyl group (Group F) at 10.47 ± 2.17 minutes, with a P-value of 0.001. Intraoperative heart rate and mean arterial blood pressure were also significantly lower in Group D than in Group F. Visual analogue scale (VAS) pain scores were significantly lower in Group D compared to Group F upon arrival at the post-anesthesia care unit and at 2 hours postoperatively (P-value < 0.05). Additionally, the morphine dose consumed in the first 12 hours after surgery was significantly lower in Group D (5.75 ± 2.20 mg) compared to Group F (8 ± 2.38 mg), with a P-value of 0.001.

**Conclusions:**

For morbidly obese patients undergoing laparoscopic sleeve gastrectomy, dexmedetomidine (DEX) proves to be an effective anesthetic choice. It not only reduces extubation time but also lowers early postoperative visual analogue scale (VAS) pain scores and opioid consumption within the first 12 hours following surgery.

## 1. Background

The rising global prevalence of obesity adversely impacts life expectancy and quality. Obesity is associated with several comorbidities, including cancer, cardiovascular diseases, hypertension, stroke, joint pain, and diabetes mellitus (1, 2). Sleeve gastrectomy (SG) has gained popularity as an effective surgical option for obesity management, known for significant and sustainable weight loss and improvement or resolution of metabolic comorbidities (3). 

Laparoscopic surgery, preferred over open surgery, reduces invasiveness, wound infections, and postoperative pain (4). However, pneumoperitoneum—the insufflation of CO_2_ into the abdomen—can stress the cardiovascular system, decreasing cardiac output and increasing both pulmonary and systemic vascular resistance, leading to higher arterial blood pressure and heart rate (5).

Opioids, commonly used for hemodynamic stability and stress reduction during surgery, are associated with adverse effects such as respiratory depression, drowsiness, vomiting, and nausea. Therefore, there is a need for alternative analgesics that better suit obese patients' perioperative management (6). Dexmedetomidine (DEX), an α2 adrenergic receptor agonist, offers a reduction in cardiac output, heart rate, and arterial blood pressure through its sympatholytic properties, making it a valuable analgesic and anesthetic-sparing agent, thereby enhancing recovery quality (7, 8).

## 2. Objectives

Our research aimed to compare the effects of fentanyl and dexmedetomidine (DEX) on intraoperative hemodynamics, recovery, and postoperative analgesic quality in morbidly obese patients undergoing laparoscopic sleeve gastrectomy (SG). We primarily focused on extubation time, with secondary outcomes including intraoperative hemodynamic stability, postoperative visual analogue scale (VAS) pain scores, total morphine consumption within the first 12 hours post-surgery, and the incidence of postoperative respiratory complications.

## 3. Methods

This randomized, double-blind, controlled trial was conducted at Helwan University Hospitals from May 2021 to May 2023. The study included 64 patients, both male and female, aged 20 to 50 years, with a BMI ≥ 35 kg/m^2^ and classified as ASA physical status II-III, who underwent laparoscopic sleeve gastrectomy (LSG). The study received approval from the Institutional Ethics Committee (approval code 26-2020) and was registered in the clinical trials registry (ID: NCT06052111). All participants provided signed informed consent. Exclusion criteria included allergy to α2-adrenergic agonists, kidney, liver, neuromuscular disorders, cardiac disease, or current opioid medication use.

Participants were randomly assigned into two groups (32 patients each) using computer-generated numbers and sealed opaque envelopes. The DEX group (Group D) received an intravenous loading dose of dexmedetomidine (Precedex, Hospira, USA, 200 μg/2 mL) at 1 μg/kg over 15 minutes prior to anesthesia induction, followed by 10 ml of 0.9% sodium chloride over 60 seconds during anesthetic induction. Post-intubation, DEX was maintained at 0.5 μg/kg/h via a syringe pump until trocar removal. The fentanyl group (Group F) received a volume-matched saline placebo over 15 minutes pre-induction and fentanyl (Fentanyl-Hamelin, Sunny pharmaceutical, Germany, 100 μg/2 mL) at 1 μg/kg diluted in 10 mL saline 0.9% IV over 60 seconds during anesthetic induction. Post-intubation, a fentanyl infusion was maintained at 1 μg/kg/hr until trocar removal.

An independent anesthetist, not involved in the study outcomes, prepared the drug solutions in identical syringes to maintain blinding; DEX was diluted to 100 μg in 50 mL saline 0.9% and fentanyl to 200 μg in 50 mL saline 0.9%, achieving similar infusion rates. Standardized anesthetic management was executed by two experienced anesthesiologists who were unaware of the specific drug administered, ensuring impartial data recording and postoperative outcome assessment.

Each patient was equipped with standard monitoring devices: An automated blood pressure cuff (NIBP), a temperature probe, a 5-lead electrocardiogram (ECG), capnography, and pulse oximetry. Baseline parameters were meticulously recorded. Drug doses were calculated based on adjusted body weight, except for atracurium, which was based on lean body weight to optimize dosing accuracy.

Preoxygenation was conducted for 3 - 5 minutes using a well-fitting face mask, ensuring optimal oxygenation before anesthesia induction. General anesthesia was initiated with lidocaine (1.5 mg/kg), propofol (1 - 2 mg/kg), and atracurium (0.5 mg/kg) to facilitate a smooth induction and intubation process. Intubation was performed with an appropriately sized cuffed endotracheal tube (ETT), which was secured and position confirmed through auscultation, observing chest expansion, and the presence of consistent capnogram waves. Additionally, all pressure points were adequately padded to prevent pressure sores during surgery, enhancing patient safety and comfort.

The lungs were ventilated in a volume-controlled mode, setting the tidal volume between 6 - 8 ml/kg based on the ideal body weight to optimize lung expansion while avoiding overdistension. The inspiratory to expiratory ratio was fixed at 1: 2, and the respiratory rate was finely adjusted to maintain normocapnia, ensuring adequate gas exchange. Positive end-expiratory pressure (PEEP) was set between 5 - 10 cm H_2_O to optimize oxygenation, maintaining an SpO_2_ level of ≥ 95%.

Post-induction, all patients were administered 1 g of paracetamol IV, 8 mg of dexamethasone IV, and a 60 mg ketorolac IV infusion to manage inflammation and pain, enhancing postoperative recovery. Anesthesia was maintained using 1-minimum alveolar concentration (MAC) of isoflurane, finely adjusted to keep the mean arterial pressure (MAP) within 20% of the baseline value. Additional doses of atracurium were administered as required to ensure adequate muscle relaxation.

The pneumoperitoneum pressure was meticulously controlled between 12 - 14 mmHg to facilitate the surgical field visibility while minimizing potential hemodynamic changes. Heart rate (HR) and MAP were diligently monitored and recorded at key surgical milestones: Baseline, after loading drug dose, post-induction, post-intubation, upon trocar insertion, during insufflation, and at regular 15-minute intervals throughout the procedure until its conclusion.

In instances of hypotension, characterized by MAP falling below 60 mmHg, management included administering 5 mg of ephedrine and a 250 mL fluid bolus to restore vascular tone and blood pressure. For bradycardia, defined as a heart rate below 50 beats per minute, 0.6 mg of atropine was administered. If these interventions failed to correct the heart rate, the infusion of the study drugs was temporarily halted.

At the end of the procedure, following desufflation, 4 mg of ondansetron was administered intravenously to all patients to prevent postoperative nausea and vomiting, enhancing overall patient comfort and recovery.

At the conclusion of the suturing procedure, the administration of isoflurane was discontinued, and the muscle relaxant was reversed using neostigmine (0.05 mg/kg) and atropine (0.02 mg/kg). Extubation was performed once patients met the necessary criteria, and the duration from the cessation of anesthesia (isoflurane discontinuation) to safe extubation was recorded as the primary outcome—extubation time. Following this, patients were transferred to the post-anesthesia care unit (PACU) with oxygen support and positioned with their head up at a 45-degree angle.

In the PACU, the modified Aldrete score was assessed, and patients were discharged one hour after achieving a score greater than 8, provided they experienced no respiratory events such as bradypnea, desaturation, or apnea, and had no active vomiting. Pain intensity was monitored using a visual analogue scale (VAS) from 0 (no pain) to 10 (the worst pain imaginable). Pain assessments were made in the PACU and subsequently every two hours for the first 12 hours postoperatively. Patients reporting a VAS score of 4 or higher received IV morphine at a dose of 2 mg, which could be repeated every 15 minutes until the pain level reduced to below 4. The timing of the first analgesic request and the total amount of opioid consumed during the first 12 hours postoperatively were also recorded, along with the incidence of postoperative respiratory complications, including apnea and hypoxemia.

### 3.1. Sample Size Justification

The estimation of sample size was conducted using G*Power 3.1.9.2, a software developed by Universitat Kiel in Germany. According to a prior study (9), an effect size of 0.78 was revealed. To achieve a statistical power of 80% and maintain a significance level (α) of 0.05, a minimum sample size of 27 patients in each group was determined using a two-tailed t-test. An additional 20% was included to account for the number of individuals who did not complete the study. A total of 32 patients were enrolled in each group.

### 3.2. Statistical Analysis

The data analysis for this study was carried out using SPSS v27 (IBM, Armonk, NY, USA). Normality of data distribution was assessed through histograms and the Shapiro-Wilks test. Quantitative parametric data were expressed as mean ± SD and analyzed using unpaired Student *t*-tests. Qualitative variables, presented as frequencies and percentages, were examined using the chi-square or Fisher's exact test. A P-value of less than 0.05 for a two-tailed test was considered statistically significant.

## 4. Results

**Figure 1.**
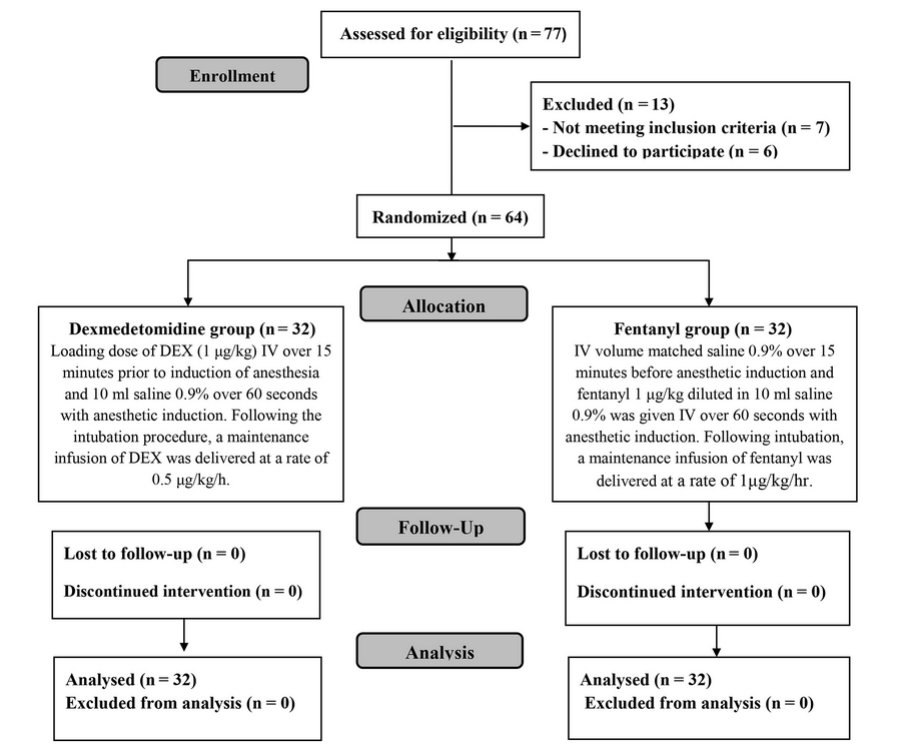
CONSORT flowchart of the enrolled patients

In this research, the eligibility of 77 patients was evaluated; 7 patients were not eligible, and 6 declined to participate in the trial. The remaining patients were randomized into two groups (32 patients each). All patients were statistically analyzed and followed up (Figure 1). 

The duration of the operation and demographic data were comparable between the two groups (Table 1). 

**Table 1.** Duration of Surgery and Demographic Data of the Studied Groups

Variables	DEX Group (n = 32)	Fentanyl Group (n = 32)	P-Value
**Age (y)**	35.09 ± 8.35	34.91 ± 7.45	0.925
**Sex**			0.790
Male	11 (34.4)	10 (31.3)	
Female	21 (65.6)	22 (68.8)	
**BMI(Kg/m** ^**2**^ **)**	43.77 ± 4.67	42.63 ± 4.41	0.318
**Duration of surgery (min)**	77.09 ± 21.00	70.16 ± 10.73	0.101

Abbreviation: BMI, Body Mass Index.

^a^ Data are presented as mean ± SD or frequency (%).

**Figure 2.**
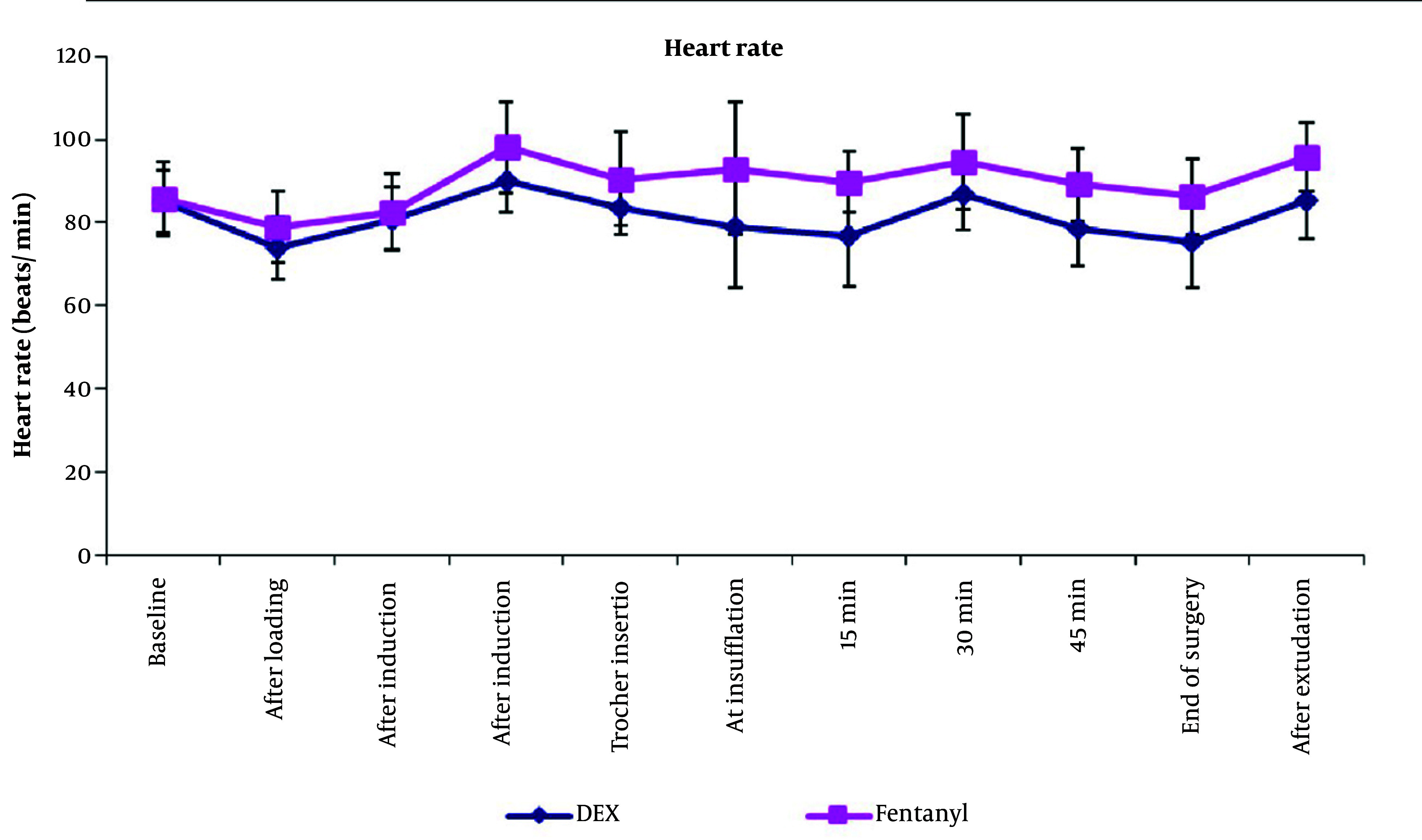
Intraoperative heart rate of both groups

Regarding hemodynamics: After the loading dose of the study drug, there was a 12.8% decrease in HR and a 13.2% decrease in MAP in group D compared to a 7.9% decrease in HR and a 4.5% decrease in MAP in group F. After intubation, HR increased by 6% with no increase in MAP values in group D, compared to a 14.4% increase in HR and an 11.5% increase in MAP values in group F. During the intraoperative period of insufflation (period of maintenance infusion of the study solutions), HR and MAP values remained below baseline value in group D, while they were above baseline values in group F. Comparing HR values between both groups, the mean baseline values of HR were insignificantly different between both groups and there was a statistically significant decrease in HR values in group D compared to group F at all times of measurement (P-value < 0.05) except HR after anesthetic induction, where there was no significant change between both groups (Figure 2). 

**Figure 3.**
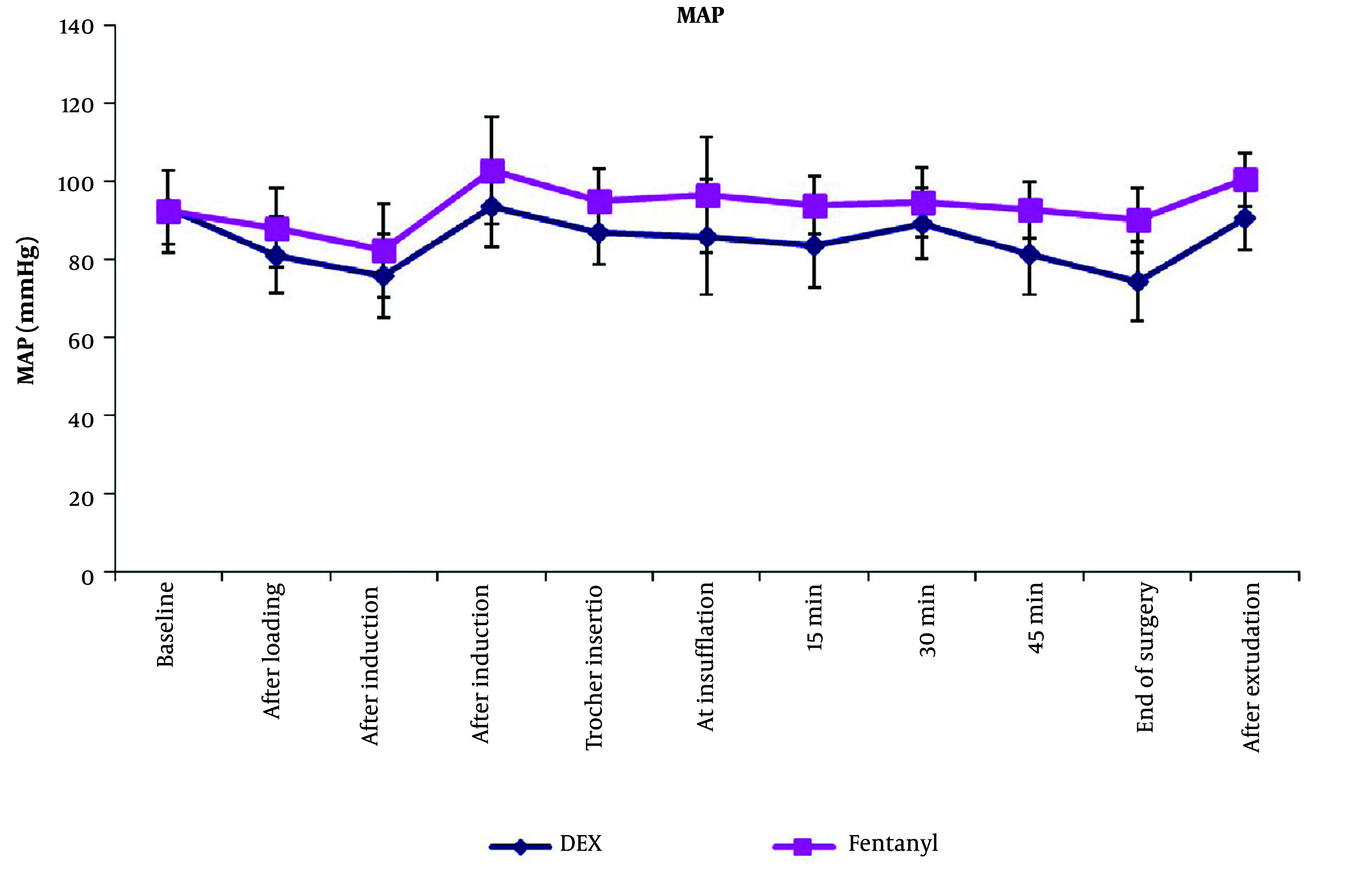
Intraoperative mean arterial pressure of both groups

Comparing MAP values between both groups, there was a statistically significant decrease in MAP values in group D compared to group F at all times of measurement except for the mean baseline values (Figure 3). 

Extubation time was significantly shorter in group D (8.25 ± 2.7 minutes) than in group F (10.47 ± 2.17 minutes), P-value = 0.001, while the PACU discharge time was comparable between both groups (Table 2). 

**Table 2.** Recovery Profile and Postoperative Analgesic Quality of Both Groups ^a^

Variables	DEX Group (n = 32)	Fentanyl Group (n = 32)	P-Value
**Time to extubation (min)**	8.25 ± 2.70	10.47 ± 2.17	0.001^b^
**Time to PACU discharge (min)**	74.06 ± 6.41	76.56 ± 8.93	0.203
**Time to 1st opioid requirement (h)**	2.13 ± 1.68	1.38 ± 1.48	0.062
**Total dose of Morphine (mg) consumption in 12 hours postoperative**	5.75 ± 2.20	8.00 ± 2.38	0.001^b^

^a^ Data are presented as mean ± SD.

^b^ Significant when P ≤ 0.05.

**Figure 4.**
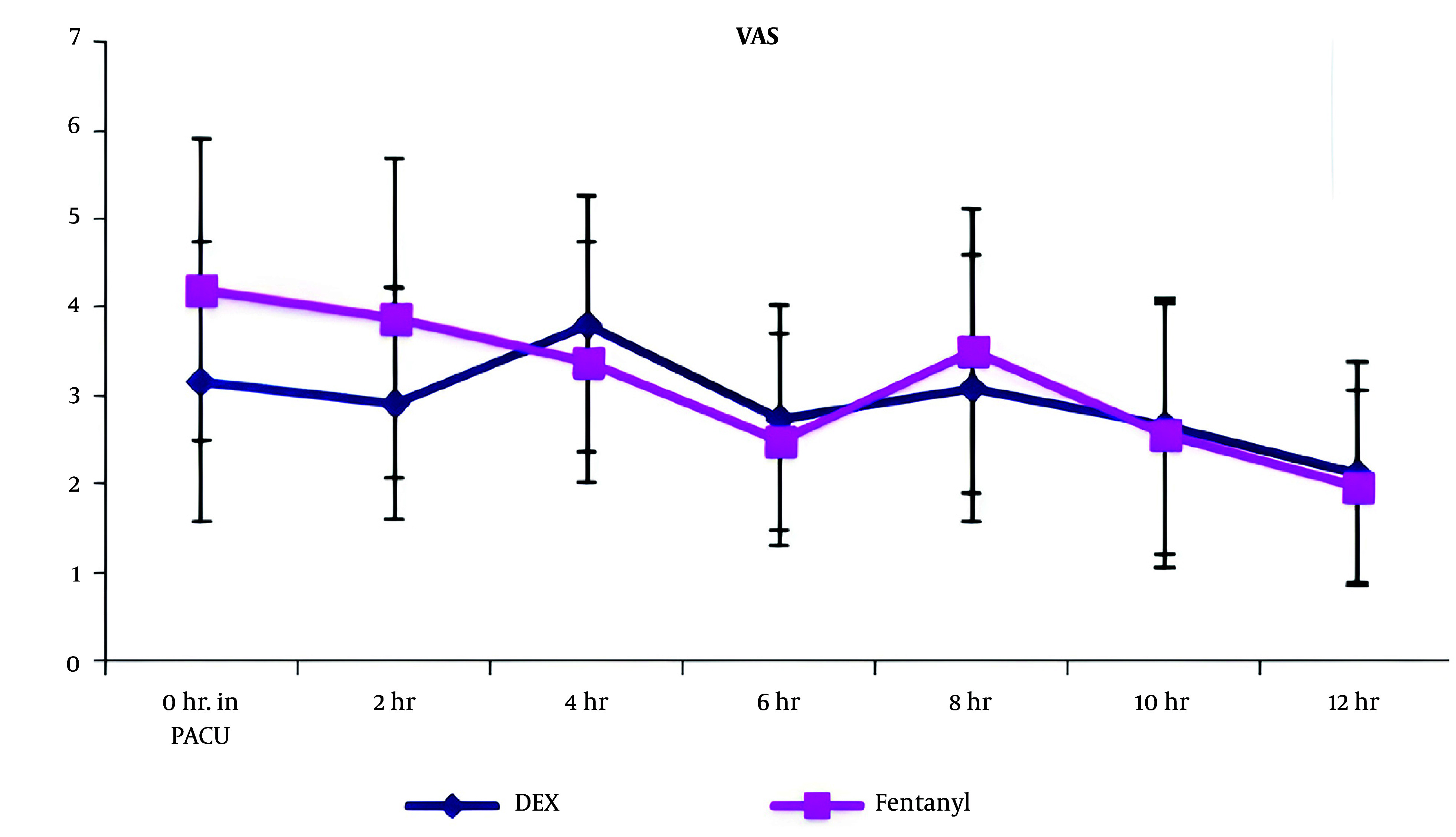
Visual analogue scale of both groups

In terms of the visual analogue score, a comparison between both groups revealed a significant decrease in VAS at PACU arrival and at 2 hours postoperatively in group D compared to group F (p value < 0.05). However, there were no statistically significant changes between both groups at 4, 6, 8, 10, and 12 hours postoperatively (Figure 4). 

Regarding the time to first opioid requirement in both groups, the mean values were 2.13 ± 1.68 hours in group D and 1.38 ± 1.48 hours in group F, showing a statistically insignificant difference between both groups (P-value = 0.062). The morphine dose consumed in the first 12 hours after surgery was significantly lower in group D (5.75 ± 2.20 mg) compared to group F (8 ± 2.38 mg), P-value = 0.001 (Table 2). 

Concerning side effects, the incidence of intraoperative bradycardia and hypotension was significantly higher in the DEX group [9 (28.1%) and 10 (31.3%)] than in the fentanyl group [2 (6.3%) and 3 (9.4%)], P values = 0.02 and 0.03, respectively. The number of ephedrine boluses did not exceed two boluses in any case of hypotension, and the study solutions were not discontinued in any case. Postoperative hypoxemia (defined as oxygen saturation less than 92% on oxygen support or less than 90% on room air) occurred in one case in the DEX group and three cases in the fentanyl group. It was adequately managed by encouraging the patient to take deep breaths and increasing the flow of inspired oxygen. No patients developed apnea in either group (Table 3). 

**Table 3.** Side Effects in Both Groups ^a^

Variables	DEX Group (n = 32)	Fentanyl Group (n = 32)	P-Value
**Bradycardia**	9 (28.1)	2 (6.3)	0.020 ^b^
**Hypotension**	10 (31.3)	3 (9.4)	0.030 ^b^
**Desaturation**	1 (3.1)	3 (9.4)	0.302
**Apnea **	0 (0)	0 (0)	-

^a^ Data are presented as frequency (%).

^b^ Significant when P ≤ 0.05.

## 5. Discussion

Due to the potential significant adverse effects associated with opioids, the American Society of Anesthesiologists advocates for the reduction or limitation of narcotic administration in obese patients during the perioperative phase (10). Anesthetists are encouraged to rely on alternative analgesic modalities to decrease opioid requirements in these patients (11).

Our findings demonstrated a shorter extubation time in the DEX group compared to the fentanyl group, significantly lower visual analogue scale (VAS) pain scores at PACU arrival and 2 hours post-surgery, and significantly lower morphine consumption during the first 12 hours postoperatively in the DEX group. These results are consistent with those of Rachana et al. (5), who conducted a study on 60 patients undergoing laparoscopic gynecological surgeries, divided equally between those receiving an IV DEX 1 µg/kg loading dose followed by a continuous infusion of 0.5 µg/kg/h, and those receiving fentanyl at the same loading and maintenance doses. They reported faster extubation and recovery times in the DEX group compared to the fentanyl group.

Similarly, Goyal et al. (12) studied 60 female patients undergoing breast cancer surgery, dividing them into fentanyl and DEX groups. They observed shorter extubation times in the DEX group compared to the fentanyl group, with a p-value of less than 0.001.

Moreover, research by El Sayed et al. (9) on 56 patients scheduled for laparoscopic bariatric surgery divided the participants into two groups: group D (DEX) received IV DEX (1 µg/kg) as a loading dose, then (0.4 µg/kg/h) for maintenance, while group N (control) received saline 0.9% at the same rate and volume. Fentanyl boluses were administered in both groups in response to hemodynamic changes. Their findings demonstrated a decline in time to extubation in the DEX group compared to the saline group. Also, PACU VAS scores were significantly lower in the DEX group compared to the saline group.

In contrast to our research, Elnahla et al. (13) evaluated the efficacy of opioid-free anesthesia (OFA) versus opioid-based general anesthesia (OA) in a cohort of 60 morbidly obese patients undergoing laparoscopic cholecystectomy. The patients were divided into two equal groups: one received OA with fentanyl (1 µg/kg as a loading dose, then 1 µg/kg/h as maintenance), and the other OFA received DEX (1 µg/kg over 10 minutes, then 0.5 µg/kg/h as maintenance); ketamine and lidocaine were also given as anesthetic adjuvants and analgesics in the OFA group. Their results revealed no significant difference in extubation times and a significant decrease in VAS values and the total analgesic dose consumed on the first postoperative day in the OFA group compared to the OA group. In this study, the authors used ketamine, lidocaine, and propofol with DEX in the OFA group, which could have a synergistic effect prolonging both the extubation time and the analgesic period.

Additionally, Tripathi et al. (14) evaluated the hemodynamic effects of fentanyl and DEX infusion in patients scheduled for elective intracranial supratentorial surgeries with a Glasgow coma score of 14 or 15. This research showed that patients receiving DEX (1 µg/kg loading and 0.4 - 0.6 µg/kg/h maintenance) experienced a statistically significant longer time to extubation compared to patients receiving fentanyl (2 µg/kg loading and 1 µg/kg/h maintenance). This variance from our research may be due to different types of patients (altered GCS in some of them), different types of surgery, longer surgical duration, prolonged duration of infusion, and the fact that intraoperative fentanyl boluses were administered in both groups.

Furthermore, in the study by Soudi et al. (15), 60 patients undergoing laparoscopic bariatric surgeries were divided into two groups: One receiving traditional balanced anesthesia (TBA) with fentanyl and the other receiving opioid-free anesthesia (OFA) using dexmedetomidine (DEX) and ketamine. The results indicated that the extubation time was significantly longer in the OFA group compared to the TBA group; moreover, the postoperative pain scores were significantly reduced in the OFA group for up to 24 hours postoperatively. This extended duration of analgesia was attributed to the additive analgesic effect of ketamine when used with DEX. Unlike our study, their DEX dosing was based on total body weight (TBW), whereas we used adjusted body weight (ABW) for drug dosing.

Additionally, Ibrahim et al. (16) assessed the effects of combining loco-regional anesthesia and OFA in 103 patients scheduled for sleeve gastrectomy. The OFA group received IV ketamine and DEX at induction, followed by maintenance doses of DEX, lidocaine, and ketamine, whereas the control group received only 1µg/kg fentanyl at induction. The study found that extubation times were longer in the OFA group, which the researchers attributed to the synergistic effects of ketamine.

Moreover, Bakan et al. (17) studied 80 patients undergoing laparoscopic cholecystectomy, splitting them into two groups: One receiving OFA with DEX and lidocaine (Group DL) and the other receiving anesthesia with opioids, specifically fentanyl and remifentanil (Group RF). Their study showed no significant differences in extubation times between the two groups, which could be due to the use of remifentanil, known for its rapid offset, as opposed to the longer-acting fentanyl used in our study.

Concerning hemodynamics, our research demonstrated that DEX significantly reduced heart rate (HR) and mean arterial pressure (MAP) compared to fentanyl throughout the surgery, underscoring DEX's efficacy as a hypotensive agent. These findings are in line with those reported by Rachana et al. (5), Tripathi et al. (14), and El Sayed et al. (9), further confirming the hemodynamic stability provided by DEX during surgical procedures.

Furthermore, Greiss et al. (18) conducted a study on 82 patients scheduled for laparoscopic surgery, dividing them into two groups: One receiving dexmedetomidine (DEX) and the other fentanyl, with both drugs administered at a loading dose of 1µg/kg followed by a maintenance dose of 0.2-0.7µg/kg/h. They found significant reductions in both heart rate (HR) and mean arterial pressure (MAP) in the DEX group, which aligned with our findings (P values 0.021 and 0.022, respectively).

In contrast, a study by Siddiqui et al. (19) on 90 patients undergoing laparoscopic cholecystectomy compared the effects of DEX, used in a regimen similar to ours, and fentanyl administered at 2.0 µg/kg over 10 minutes, then maintained at 1.0 µg/kg/h. Their findings indicated no significant difference in HR between the groups, whereas MAP was significantly lower throughout most of the surgery in the fentanyl group. The differences from our study may stem from their use of total intravenous anesthesia and the type of surgery performed, which could influence the hemodynamic responses.

Salman et al. (20) also explored the hemodynamic effects in 60 patients undergoing gynecologic laparoscopic surgery, comparing those receiving remifentanil to those administered DEX. Their study reported no significant differences in hemodynamics between the two groups, highlighting the variable impact of DEX in different surgical contexts.

As for complications, our study observed a significantly higher incidence of bradycardia (HR < 50 bpm) and hypotension (MAP < 60 mmHg) in the DEX group (28.1% and 31.3%, respectively) compared to the fentanyl group (6.3% and 9.4%, respectively), with P values of 0.020 and 0.030. This increase in side effects with DEX use, particularly regarding cardiovascular stability, underscores the need for careful monitoring when using this agent. Despite these issues, we recorded no cases of apnea, and the rates of desaturation were similar between groups, suggesting that while DEX increases the risk of certain complications, it does not exacerbate respiratory risks.

Echoing our findings, the study by Soudi et al. (15) also noted a significant increase in hypotension incidents within their OFA group (DEX plus ketamine) compared to their TBA group (fentanyl), further emphasizing the cardiovascular effects of DEX and its combinations in anesthesia management.

Beloeil et al. (21) conducted a study involving 314 patients undergoing non-cardiac surgery, where participants were divided into groups receiving either a remifentanil infusion or a dexmedetomidine (DEX) infusion. Their findings showed that the DEX group experienced a significantly higher rate of bradycardia compared to the remifentanil group, with a p-value of 0.009. This aligns with observations from other studies indicating that DEX can lead to more cardiovascular stability issues such as bradycardia.

In contrast, Turgut et al. (22) explored the effects of DEX versus fentanyl in 50 patients undergoing lumbar laminectomy. They reported bradycardia in five patients in the DEX group and two in the fentanyl group, but the differences were not statistically significant. Additionally, no cases of hypertension (HTN) or hypotension were noted in either group. This variation from our findings could stem from the different surgical procedures involved, patient demographics, and the dosing regimen of DEX, which was lower in their study. Such factors can influence the incidence and severity of side effects associated with anesthetic agents, underlining the importance of considering specific clinical contexts when evaluating the safety and efficacy of anesthesia protocols.

### 5.1. Limitation of the Study

First, the assessment of postoperative Visual Analogue Scale (VAS) scores and morphine dosage was restricted to the first 12 hours following surgery. Second, our study did not evaluate the incidence of postoperative nausea and vomiting.

### 5.2. Conclusions

For morbidly obese patients undergoing laparoscopic sleeve gastrectomy (SG), Dexmedetomidine (DEX) proves to be an effective anesthetic choice as it shortens extubation time and reduces early postoperative visual analogue scale (VAS) pain scores and opioid use within the first 12 hours after surgery.

## Data Availability

The dataset presented in the study is available on request from the corresponding author during submission or after publication.

## Clinical Trial Registration Code:

NCT06052111.
